# Supplementary material for: Pharmacological intervention of behavioural traits and brain histopathology of prenatal valproic acid-induced mouse model of autism
Source: PLoS One. 2024 Sep 24;19(9):e0308632. doi: 10.1371/journal.pone.0308632 (PMC11421774; doi:10.1371/journal.pone.0308632)
Supplement: S1 Dataset — (PDF) [file pone.0308632.s001.pdf]

Table 1: Comparison between Negative Control 1 and treated groups

Tests	Parameters Checked	Group Names (Mean±SEM)		
		Negative Control 1 (Water on Autistic Mice)	Treated 1 (30mg/kg Betahistine on Autistic Mice)	Treated 2 (3mg/kg Betahistine + 0.8mg/kg Rasagiline on Autistic Mice)
Y-maze	Spontaneous Alternation Percentage %	48.5583±3.39298	59.1217±2.4581	64.2683±5.79437*
Open-field	Urination	0.17±0.167	0.17±0.167	0.33±0.211
	Defecation	1.67±0.494	2.5±0.764	1.67±0.422
	Rearing	39.17±6.085	17±2.966***	27.17±5.212
	Central Area Frequency	4.83±1.276	1.83±0.703	4±0.683
	Central Area Duration	17.67±5.487	8±3.307	11.83±2.007
	Line Crossing	315.17±19.352	216.67±21.814**	338.67±8.135
Hole-board	Line Crossing	51.83±15.145	18.83±7.547*	48.33±8.539
	Head Dipping	27.83±4.909	30.83±4.362	32±3.651
	Rearing	0.17±0.167	1.17±0.601	0.5±0.224
Hole-cross	hole-crossing	6.83±0.833	2.5±0.619**	7.83±1.195

Comparison of negative control 1 (NC1) was done with all of the other groups

*p<0.05; **p<0.01; ***p<0.001.

Table 2: Comparison between Control and treated groups

Tests	Parameters Checked	Group Names (Mean±SEM)		
		Control (Water on Healthy Mice)	Treated 1 (30mg/kg Betahistine on Autistic Mice)	Treated 2 (3mg/kg Betahistine + 0.8mg/kg Rasagiline on Autistic Mice)
Y-maze	Spontaneous Alternation Percentage %	67.6883±4.88809	59.1217±2.4581	64.2683±5.79437
Open-field	Urination	0.5±0.224	0.17±0.167	0.33±0.211
	Defecation	2.33±0.333	2.5±0.764	1.67±0.422
	Rearing	14.33±2.404	17±2.966	27.17±5.212*
	Central Area Frequency	4.17±1.352	1.83±0.703	4±0.683
	Central Area Duration	13.67±4.432	8±3.307	11.83±2.007
	Line Crossing	188±12.759	216.67±21.814	338.67±8.135***
Hole-board	Line Crossing	35.83±6.71	18.83±7.547	48.33±8.539
	Head Dipping	26.67±5.226	30.83±4.362	32±3.651
	Rearing	0.5±0.342	1.17±0.601	0.5±0.224
Hole-cross	hole-crossing	1.67±0.919	2.5±0.619	7.83±1.195***

Comparison of Control (C) was done with all of the other groups

**p*<0.05; ***p*<0.01; ****p*<0.001.

Table 3: Comparison of Negative Control group with the treated groups (T3, T4 and T5)

Test	Parameters Checked	Group Name (Mean ± SEM)			
		Negative Control (Water in autistic mice)	T3 (0.3mg/kg Donepezil Hydrochloride Monohydrate on autistic mice)	T4 (1mg/kg Donepezil Hydrochloride Monohydrate on autistic mice)	T5 (0.6mg/kg Donepezil Hydrochloride Monohydrate on autistic mice)
Open Field	Urination	.40 ± .245	.00 ± .000	.33 ± .211	.20 ± .200
Open Field Hole Board	Defecation	.20 ± .200	.17 ± .167	.50 ± .224	.60 ± .400
	Rearing	16.80 ± 6.320	12.00 ± 3.759	.50 ± .224**	5.60 ± 2.731*
	Center Area Frequency	2.60 ± 1.661	1.67 ± .615	.00 ± .000*	.60 ± .400
	Line Crossing	166.00 ± 42.653	166.33 ± 38.473	3.83 ± .833***	56.80 ± 23.064*
	Head Dipping	8.80 ± 3.639	9.50 ± 3.149	1.33 ± .989*	2.00 ± .837
Hole Board Hole Cross	Stool	1.00 ± .316	.50 ± .224	.17 ± .167	.60 ± .245
	Area	10.60 ± 4.885	18.50 ± 7.886	13.67 ± 3.159	1.20 ± .490
	Hole Crossing	2.40 ± 2.159	.83 ± .543	.67 ± .667	.40 ± .400

Results were presented as mean ± standard error of means.

* $p<0.05$; ** $p<0.01$; *** $p<0.001$.

Table 4: Comparison of Control group with the treated groups (T3, T4 and T5)

Test	Parameters Checked	Group Name (Mean ± SEM)			
		Control (Water in healthy mice)	T3 (0.3mg/kg Donepezil Hydrochloride Monohydrate on autistic mice)	T4 (1mg/kg Donepezil Hydrochloride Monohydrate on autistic mice)	T5 (0.6mg/kg Donepezil Hydrochloride Monohydrate on autistic mice)
Open Field	Urination	.17 ± .167	.00 ± .000	.33 ± .211	.20 ± .200
	Defecation	.83 ± .477	.17 ± .167	.50 ± .224	.60 ± .400
	Rearing	5.83 ± 2.272	12.00 ± 3.759	.50 ± .224	5.60 ± 2.731
	Center Area Frequency	1.00 ±.683	1.67 ± .615	.00 ± .000	.60 ± .400
	Line Crossing	72.67 ± 25.831	166.33 ± 38.473	3.83 ± .833	56.80 ± 23.064
Hole Board	Head Dipping	14.00 ± 2.720	9.50 ± 3.149	1.33 ± .989	2.00 ± .837
	Stool	1.00 ± .516	.50 ± .224	.17 ± .167	.60 ± .245
	Line Crossing	22.83 ± 8.631	18.50 ± 7.886	13.67 ± 3.159	1.20 ± .490*
Hole Cross	Hole Crossing	1.50 ± 1.118	.83 ± .543	.67 ± .667	.40 ± .400

Results were presented as mean ± standard error of means.

* $p<0.05$; ** $p<0.01$; *** $p<0.001$.
