# Supplementary material for: Pharmacological intervention of behavioural traits and brain histopathology of prenatal valproic acid-induced mouse model of autism
Source: PLoS One. 2024 Sep 24;19(9):e0308632. doi: 10.1371/journal.pone.0308632 (PMC11421774; doi:10.1371/journal.pone.0308632)
Supplement: Attachment — Submitted filename: Comments PONE-D-23-30357.docx [file pone.0308632.s002.docx]

1. The authors chose to treat the rats from PND44 to 64. Why this particular time window, frequency and duration of treatment? What is the authors' hypothesis when treating after weaning, when the neurodevelopmental period is mostly completed? Of note, relieving effects of treatment observed in nearly young adults are encouraging.
2. The term ‘Alteration’ and ‘alternation’ is written interchangeably in Y maze in methods and results section. It should be spontaneous alternation and not alteration. Correct it.
3. Data representation: histograms with scatter plots are highly recommended to allow the reader to assess the animal numbers per group and the dispersion of individual data at a glance.
4. Autistic mice: avoid using such wording; use the terms "VPA-exposed mice" instead.
5. In abstract it is mentioned that “After administration of the experimental doses, various locomotor tests: Open Field, Hole-Board, Hole Cross tests and behavioural tests: Y-Maze Spontaneous Alternation Test were performed and compared with positive and negative control groups”. What is the positive control group that is mentioned in this statement, which is nowhere mentioned in the rest of manuscript?
6. What is the significance of combining betahistine and rasagiline, why were both the drugs given in combination in T1 group? Kindly add the significance in the manuscript.
7. Authors have concluded that drugs have potential to ameliorate short-term memory deficits, it is recommended to perform other behavioral parameters for memory assessment like, Morris water maze, Radial Arm maze and Novel object recognition, to have better glance into the cognitive outcomes.
8. Furthermore it is recommended to report the histopathological changes in the appropriate regions of the brain of VPA exposed mice, using hematoxylin and eosin stain and Nissl stain, and also estimation of levels of acetyl choline and histamine in the VPA exposed mice and treated mice is highly recommended. These basic investigations will make the preliminary findings from this study more reliable and convincing for further research into these molecules and their mechanisms in ASD.
