# Supplementary material for: Pharmacological intervention of behavioural traits and brain histopathology of prenatal valproic acid-induced mouse model of autism
Source: PLoS One. 2024 Sep 24;19(9):e0308632. doi: 10.1371/journal.pone.0308632 (PMC11421774; doi:10.1371/journal.pone.0308632)
Supplement: Attachment — Submitted filename: Response to Reviewers.docx [file pone.0308632.s003.docx]

**Journal requirements:**

**When submitting your revision, we need you to address these additional requirements.**

**1. Please ensure that your manuscript meets PLOS ONE's style requirements, including those for file naming. The PLOS ONE style templates can be found at**

**Response:** We have made sure all the PLOS ONE’S style requirements for fie naming have been followed throughout the manuscript.

**2. We note that Figure 2 in your submission contains copyrighted images. All PLOS content is published under the Creative Commons Attribution License (CC BY 4.0), which means that the manuscript, images, and Supporting Information files will be freely available online, and any third party is permitted to access, download, copy, distribute, and use these materials in any way, even commercially, with proper attribution. For more information, see our copyright guidelines:** [**http://journals.plos.org/plosone/s/licenses-and-copyright**](http://journals.plos.org/plosone/s/licenses-and-copyright)**.**

**We require you to either (1) present written permission from the copyright holder to publish these figures specifically under the CC BY 4.0 license, or (2) remove the figures from your submission:**

**1. You may seek permission from the original copyright holder of Figure 2 to publish the content specifically under the CC BY 4.0 license.**

**We recommend that you contact the original copyright holder with the Content Permission Form (**[**http://journals.plos.org/plosone/s/file?id=7c09/content-permission-form.pdf**](http://journals.plos.org/plosone/s/file?id=7c09/content-permission-form.pdf)**) and the following text:**

**“I request permission for the open-access journal PLOS ONE to publish XXX under the Creative Commons Attribution License (CCAL) CC BY 4.0 (**[**http://creativecommons.org/licenses/by/4.0/**](http://creativecommons.org/licenses/by/4.0/)**). Please be aware that this license allows unrestricted use and distribution, even commercially, by third parties. Please reply and provide explicit written permission to publish XXX under a CC BY license and complete the attached form.”**

**Please upload the completed Content Permission Form or other proof of granted permissions as an "Other" file with your submission.**

**In the figure caption of the copyrighted figure, please include the following text: “Reprinted from [ref] under a CC BY license, with permission from [name of publisher], original copyright [original copyright year].”**

**2. If you are unable to obtain permission from the original copyright holder to publish these figures under the CC BY 4.0 license or if the copyright holder’s requirements are incompatible with the CC BY 4.0 license, please either i) remove the figure or ii) supply a replacement figure that complies with the CC BY 4.0 license. Please check copyright information on all replacement figures and update the figure caption with source information. If applicable, please specify in the figure caption text when a figure is similar but not identical to the original image and is therefore for illustrative purposes only.**

**Response:** Since we did not incorporate figures for any neuro-behavioural test, therefore, we have removed Figure 2 to avoid copyrighting issues.

**Response to the reviewers' comments:

Reviewer #1:**

- A Figure is needed to show the affinities and selectivity of test compound betahistine.

**Response:** Thank you for your suggestion. This study does not focus on the molecular mechanism of betahistine. Our aim was to study the behavioural mechanism of betahistine on VPA-induced mice. The affinity and selectivity of betahistine has already been established and information regarding its binding affinity and selectivity has been referred to in the revised manuscript in line 392-394 with reference 43. On our next project we are considering studying the molecular mechanism of betahistine over its behavioural pattern.

- When discussing the involvement of histaminergic and/or cholinergic neurotransmission in the observed effects for betahistine, cite the following significant studies in your discussion;

1) Eissa N, Al Awad M, Venkatachalam K, Jayaprakash P, Thomas SD, Zhong S, Stark H, Sadek B. Simultaneous antagonism at H3R/D2R/D3R reduces autism-like self-grooming and aggressive behaviours by mitigating MAPK activation in mice. Int. J. Mol. Sci. 2023; 24:526.

2) Saad AK, Akour A, Mahboob A, AbuRuz S, Sadek B. Role of brain modulators in neurodevelopment: focus on autism spectrum disorder and associated comorbidities. Pharmaceuticals 2022, 15:612.

3) Eissa N, Venkatachalam K, Jayaprakash P, Falkenstein M, Frank A, Reiner-Link D, Stark H, Sadek B. The multi-targeting ligand ST-2223 with histamine H3 receptor and dopamine D2/D3 receptor antagonist properties mitigates autism-like repetitive behaviours and brain oxidative stress in mice. Int. J. Mol. Sci. 2021; 22(4), 1947.

4) Eissa N, Azimullah S, Jayaprakash P, Jayaraj RL, Reiner D, Ojha SK, Beiram R, Stark H, Łażewska D, Kieć-Kononowicz K, Sadek B. The dual-active histamine H3 receptor antagonist and acetylcholine esterase inhibitor E100 alleviates autistic-like behaviours and oxidative stress in valproic acid induced autism in mice. Int. J. Mol. Sci. 2020; 21(11), 3996.

**Response:** Thank you for your suggestion. We have cited the aforementioned studies in our discussion.

Reference 01 can be found as reference 41 in line number: 384,406, 416, 453, and 459.

Reference 02 can be found as reference 46 in line number: 416 and 418.

Reference 03 can be found as reference 14 in line number: 67, 87, 389, and 446.

Reference 04 can be found as reference 24 in line number: 111, 122, 353, and 358.

- References to justify the selected and used doses of all test compounds are missing.

**Response:** Thank you for your comment. The doses were reviewed from the referenced articles. Some doses were modified, which are justified in the discussion section. Betahistine and Rasagiline doses were justified in line 446-455 using reference no 33,36, 41 & 48 in the manuscript. Donepezil doses were justified in line 456-459 using reference no 41,49 & 50 in the manuscript.

- Quality of all Figures should be improved, as they are difficult to read.

**Response:** Thank you for your suggestion. The quality of all the figures have now been improved.

**Reviewer #2:**

1. The authors chose to treat the rats from PND44 to 64. Why this particular time window, frequency and duration of treatment? What is the authors' hypothesis when treating after weaning, when the neurodevelopmental period is mostly completed? Of note, relieving effects of treatment observed in nearly young adults are encouraging.

**Response**: Your query is appreciated. The time window selected for dosage and performing of behavioural studies were done according to the referenced article number 23 of our manuscript.

The dosages are administered after weaning and not at a young age.

2. The term ‘Alteration’ and ‘alternation’ is written interchangeably in Y maze in the methods and results section. It should be spontaneous alternation and not alteration. Correct it.

**Response:** Thank you for your notification. Recommended changes have been made accordingly in the line numbers 215 & 425.

3. Data representation: histograms with scatter plots are highly recommended to allow the reader to assess the animal numbers per group and the dispersion of individual data at a glance.

**Response:** We appreciate this valuable suggestion. In this manuscript, the quality of the existing figures have been improved according to the other two reviewer’s suggestion. In addition, as per your suggestion, the number of animals per group have been mentioned in the method section. However, we do agree with you that providing histograms with scatter plots would be better, but since the error margin within the sample population is very low, we opted for bar graph for better representation of our data and easier understanding of the overall effect. Furthermore, we can surely provide the individual data for the mice.

4. Autistic mice: avoid using such wording; use the terms "VPA-exposed mice" instead.

**Response:** Thank you for your notification. Changes have been made accordingly. (Line- 26, 30, 63, 81, 337, 342, and 429)

5. In abstract it is mentioned that “After administration of the experimental doses, various locomotor tests: Open Field, Hole-Board, Hole Cross tests and behavioural tests: Y-Maze Spontaneous Alternation Test were performed and compared with positive and negative control groups”. What is the positive control group that is mentioned in this statement, which is nowhere mentioned in the rest of the manuscript?

**Response:** Thank you for your reply. In Line 23 the control group is mentioned as 'positive control'. The word has been changed to control instead of positive control.

6. What is the significance of combining betahistine and rasagiline, why were both the drugs given in combination in the T1 group? Kindly add the significance in the manuscript.

Response: Thank you for your query. The significance of combining betahistine and rasagiline has been mentioned in line 400-406.

7. Authors have concluded that drugs have potential to ameliorate short-term memory deficits, it is recommended to perform other behavioural parameters for memory assessment like, Morris water maze, Radial Arm maze and Novel object recognition, to have a better glance into the cognitive outcomes.

**Response:** We are grateful for the suggestion you have provided. We did not mention about these tests in the ethical permission, hence it is not possible for us to conduct these experiments. Moreover, we have already sacrificed the mice for histopathological data. We will consider your valuable suggestions for future studies.

8. Furthermore it is recommended to report the histopathological changes in the appropriate regions of the brain of VPA-exposed mice, using hematoxylin and eosin stain and Nissl stain, and also estimation of levels of acetylcholine and histamine in the VPA-exposed mice and treated mice is highly recommended. These basic investigations will make the preliminary findings from this study more reliable and convincing for further research into these molecules and their mechanisms in ASD.

**Response:** Thank you for this important suggestion. Now we have incorporated the findings of histopathology which, we believe, have improved the quality of the study.

**Reviewer #3:**

1. In Lines 23 and 26 leave space after ‘p’ to be consistent. Apply this to the results section too.

**Response:** Thank you for your notification. Recommended changes have been made accordingly in line 24 and 27. For consistency, we have checked the whole manuscript carefully.

2. Sometimes the authors mentioned autism and other times ASD. Please be consistent.

**Response:** Thank you for your notification. Recommended changes have been made throughout the manuscript, accordingly.

3. Why were the 2 mice genders used? Will this not have any impact on the behavior results?

**Response:** Thank you for your valuable comment. There was a shortage of VPA-induced mice pups to conduct the whole experiment with only one specific gender since some of the VPA- induced mother mice died before giving birth. Hence, we have compared the male treated groups with male control groups. Similarly, the female treated groups were compared with female control groups. We did not draw a contrast between the male or female groups.

4. I suggest that the second group of healthy offspring from the healthy mothers (NC2) to be Betahistine dihydrochloride of 3mg/kg in combination with 0.8mg/kg of Rasagiline to ensure that there are no off-target effects due to the combination of the drugs. This is important as especially there is no absence of in vitro profile of the combination of these drugs. Why is the current NC2 used?

**Response:** We extend our gratitude for your input. Both Rasagiline and Betahistine have passed clinical trials and their safety is not in question. Moreover, due to the shortage of mice pups this group was avoided.
To negate the effects of rasagiline on VPA-induced mice we have used 0.8mg/kg of rasagiline on healthy mice as the negative control 2 group (NC2). The NC2 group helps us to understand if any derogatory effects arose from the administration of Rasagiline.

5. Is there any results for the effects of Rasagiline only in VPA-induced mouse of ASD on all behavioral tests studied?

**Response:** We thank you for your query. We did not test the effects of 0.8mg/kg of Rasagiline only in VPA-induced mice. Sufficient VPA-injected mother mice did not survive to give birth to the required number of mice pups to make this VPA-induced group. An explanation of which has now been added in the methodology section. Moreover, the project did not aim to evaluate MAO-B Rasagiline’s effect separately on VPA induced mice. Furthermore, we have referenced a study that reported 0.8mg/kg of rasagiline alone could not significantly give any neuroprotective effects in transgenic mice models of neurodegeneration in line 401-404, reference 37.

6. Line 343 mentions ‘In the study evidence in support of the claim was observed in the Negative control 2 group (NC2) whereby no significant change in short-term memory retention when compared with the Control group (C). To test for the neuroprotective effect of rasagiline, the effect of rasagiline only should be tested on an ASD mouse model of ASD. This group is missed.

**Response:** Thank you for your suggestion. Rasagiline of 0.8mg/kg was added to T1 group to counteract the tolerance initiated by 3mg/kg of Betahistine in subjects after 8 days of administration, which has been discussed in the manuscript line 385-388 (Reference 2, 15, 42). The experiment did not aim to evaluate MAO-B inhibitor rasagiline’s effect on VPA induced mice. Hence this group was avoided. We will consider it for future studies.

7. The quality of all the figures is poor. Figures with high resolution need to be added.

**Response:** Thank you for your suggestion. The quality of the figures have been improved now as per the suggestion.

8. In Figure 1, add the full name of the behavioral tests or mention them in the figure caption.

**Response:** Thank you for your notification. We have added them accordingly.

9. In Line 223, 224, and 343 the control and negative control 1 should all be in small letters as it is throughout the paper.

**Response:** Thank you for your notification. We have changed it throughout the manuscript.

10. Elaboration is required on how betahistine showed anxiolytic and donepezil showed anxiogenic properties at different doses.

**Response:** Thank you for your suggestion. Since the focus of the study was to evaluate the behavioural trait of two drugs on VPA-induced mouse model, it is hard to make any comment about the anxiolytic and anxiogenic effect of the drugs by conducting only few neuro-behavioural tests. That is why, we have rephrased the sentence to better reflect our thought with regards to the experiments that we have carried out.

11. In Line 286 remove the bracket.

**Response:** Thank you for your comment. Bracket has been removed as per the advice.

12. In Lines 292 and 292 ‘embryonic’ should be in small letters.

**Response:** Thank you for your comment. Recommended changes have been made accordingly.

13. In 294 remove the extra space.

**Response:** Thank you for your notification. Extra space has been removed.

14. The authors should mention VPA-induced mice, not valproic acid-induced mice throughout the paper. The abbreviations should be described the first time in the text only.

**Response:** Thank you for your suggestion. We have changed it throughout the manuscript.

15. In line 404, rephrase the sentence. There are no types of ASD patients.

**Response:** Thank you for your insight. We have changed accordingly.

16. Please check the manuscript for the English language.

**Response:** We have checked the manuscript for the English language.
